# Using machine learning algorithms to identify genes essential for cell survival

**DOI:** 10.1186/s12859-017-1799-1

**Published:** 2017-10-03

**Authors:** Santosh Philips, Heng-Yi Wu, Lang Li

**Affiliations:** Center for Computational Biology and Bioinformatics, Indiana University, 410 West 10th Street, HITS 5003 lab, Indianapolis, IN 46202 USA

**Keywords:** Machine learning, Gene essentiality, Literature mining

## Abstract

**Background:**

With the explosion of data comes a proportional opportunity to identify novel knowledge with the potential for application in targeted therapies. In spite of this huge amounts of data, the solutions to treating complex disease is elusive. One reason being that these diseases are driven by a network of genes that need to be targeted in order to understand and treat them effectively. Part of the solution lies in mining and integrating information from various disciplines. Here we propose a machine learning method to mining through publicly available literature on RNA interference with the goal of identifying genes essential for cell survival.

**Results:**

A total of 32,164 RNA interference abstracts were identified from 10.5 million pubmed abstracts (2001 - 2015). These abstracts spanned over 1467 cancer cell lines and 4373 genes representing a total of 25,891 cell gene associations. Among the 1467 cell lines 88% of them had at least 1 or up to 25 genes studied in a given cell line. Among the 4373 genes 96% of them were studied in at least 1 or up to 25 different cell lines.

**Conclusions:**

Identifying genes that are crucial for cell survival can be a critical piece of information especially in treating complex diseases, such as cancer. The efficacy of a therapeutic intervention is multifactorial in nature and in many cases the source of therapeutic disruption could be from an unsuspected source. Machine learning algorithms helps to narrow down the search and provides information about essential genes in different cancer types. It also provides the building blocks to generate a network of interconnected genes and processes. The information thus gained can be used to generate hypothesis which can be experimentally validated to improve our understanding of what triggers and maintains the growth of cancerous cells.

**Electronic supplementary material:**

The online version of this article (10.1186/s12859-017-1799-1) contains supplementary material, which is available to authorized users.

## Background

There is no lack for data or scientific literature as they continue to grow at an exceedingly exponential rate; yet there is this unquenchable thirst for knowledge. The knowledge that can lead to new discoveries, aid in making clinical decisions and designing efficient therapeutic strategies are hidden within this huge mass of data and literature. It has been shown decades earlier that the medical literature holds hidden knowledge that can be exploited in treating complex diseases [1–6]. In spite of the availability of this huge amounts of literature two thirds of the questions that clinicians raise about patient care in their practice remain unanswered [7]. These question most often could be classified into a small set of generic questions [8] but require a diverse set of answers based on the clinicians specialty. With the advances in technology and the completion of the human genome we have data, but the challenge lies in how to identify the crucial knowledge that can lead to a better understanding of the disease pathology and equip the clinician to make informed decisions as to the best course of therapeutic action. In addition the various factors that can influence or contribute to disease susceptibility or progression poses a challenge to scientist in finding a preventative or therapeutic solution for these diseases [9–11]. The challenges in finding a cure are proportionally increasing with complexity presented by the disease. The question most commonly asked when dealing with huge amounts of data is, how the low value data can be transformed to high value knowledge which can then be applied to treating complex diseases more effectively. There is no lack for data, but connecting the information across diverse disciplines is challenging [12–14].

The heterogeneous nature of the scientific literature across multiple disciplines is something that can be exploited to identify crucial knowledge that underlies the essence of survival. The free availability of this unstructured text makes it the biggest and most widely used for the identification of new knowledge. It would be highly impossible for a human to devour this huge amount of literature to identify the dots that connect various components within a pathway that can be targeted to effectively treat a disease, especially when the information is present in non-interacting articles. Manual curation is a possibility with the advantage of being accurate, but comes at a high cost of time, labor and finding expertise in multiple disciplines. The use of computers and more specifically machine learning algorithms that can be trained to identify relevant literature and then extract the relationships between entities of interest to produce clinically applicable knowledge is gaining popularity in the race to find cures. The later though highly scalable with the ever increasing growth of literature is error prone due to the complexity of natural languages used. The ultimate goal of information access is to help the user or practitioner in finding relevant documents that satisfy their information needs so they can gain wisdom and apply it to their practice. The challenge still remains; how can we effectively use the tools and resources in finding wisdom from the huge amounts data.

RNA interference is a very powerful biological process that involves the silencing of gene expression in eukaryotic cells [15–20]. It is indeed a natural host defense mechanism by which exogenous genes, such as viruses are degraded [21–23]. With the emergence of the RNA interference technology, scientist have been able to study the consequences of depleting the expression of specific genes that code for pathological proteins and are able to observe the resultant cellular phenotypes, which can provide insights into the significance of the gene. Diseases that are associated or driven by genes, such as cancer, autoimmune disease and viral disease can take advantage of RNA interference to generate a new class of therapeutics. Synthetic RNAi can be developed to trigger the RNA interference machinery to produce the desired silencing of genes [24–26]. The power of this process can be harnessed to identify and validated drug targets and also in the development of targeted gene specific medicine.

One of the benefits of RNA interference technology, is that it provides information about the function of genes within an organism and helps us in identifying essential genes. Essential genes are those that are very important towards the survival of a cell or organism [27]. Identification of the minimum essential genes required for a cell to survive and being able to generate distinct sets that can represent normal versus cancer cell survival will not only enhance our understanding on what causes a normal cell to progress into a cancerous cell but will also provide the precise location of the gene that is the driving force of uncontrolled cell proliferation. This crucial knowledge can guide in the development of targeted cancer treatments. For example, it is very evident today, that breast cancer is no longer a single disease but heterogeneous in nature requiring different prognosis and treatments [28–31]. Since tumors are highly heterogeneous in nature, there may be more than one gene that needs to be targeted within the heterogeneous population of cells, which makes the treatment of cancer so complex. By identifying these essential genes, one can use them as building blocks to capture the heterogeneity of the tumor environment and improve the clinical decision making in treating them more effectively and with precision.

In our study, through the use of text mining and machine learning algorithms, we were able to scan through 10.5 M abstract and retrieve those relevant to RNAi studies. We were able to identify the genes that are essential for cell survival. Given the heterogeneous nature of complex disease, our study reveals the power of mining literature that can be harnessed to generate hypothesis leading to novel targeted clinical applications.

## Methods

### Abstract selection and corpus construction

The Medline database was queried for abstracts that studied the effects of siRNA or drugs on cell lines using the following boolean query structure [(siRNA or shRNA or drug) AND (cell line name)] across 6 different cell lines, namely MCF7, MCF10A, SKBR3, HS578T, BT20, and MDAMB231 The resultant PMIDs of the query were converted to XML and parsed to extract the PMID, article title and article abstract. These files formed the initial unfiltered set of abstracts and were converted to a pdf format to aid in the manual process of scanning them to select the most relevant abstracts to construct the text corpus. In addition these abstracts were further divided among four other individuals consisting of a high school student and three master’s level students for manual scanning and classification. The abstracts were read and then grouped under four categories as follows:

i. RNAi: These abstracts had siRNA/shRNA being studied, along with the cell line used and the resultant cell phenotype.
ii. Drug: These abstracts had a drug being studied, along with the cell line used and the resultant cell phenotype.
iii. Drug-Drug: These abstracts had a drug interaction being studied, along with the cell line used and the resultant cell phenotype.
iv. NA (Not Applicable): If the abstract did not fall into any of the above categories it was labelled as NA.

For an abstract to be placed in any of the categories (i) – (iii) they needed to have all three components, namely siRNA or drug and cell line and resultant cell phenotype. If one of these components were not clearly stated or was missing, the abstract was placed in the NA category. Close to 2000 abstracts were manually screened using the above criteria.

### Training and testing datasets

The abstracts from the above classification were converted to individual text files and used to create the positive and negative classes namely RNAi and Non_RNAi. The training and testing datasets consisted of various combinations as shown in the Table 1. The text files representing the training and testing datasets were converted into the WEKA native file format, namely ARFF (attribute relation file format) using the java TextDirectoryLoader class. The final training set consisted of 120 RNAi abstracts in the RNAi class and a total of 1700 abstracts from drug, drug-drug, NA and RNS in the Non_RNAi class. The testing set consisted of 101 RNAi abstracts in the RNAi class and a total of 1700 abstracts from drug, drug-drug, NA and RNS in the Non_RNAi class.

**Table 1:** Composition of the training and testing sets used to test the various weka classifiers

Set	Training		Testing		Data
	Positive	Negative	Positive	Negative	
1	100	300	100	300	r,d,dd,g
2	100	100	100	100	r,d,dd,na
3	100	300	100	300	r,d,dd,na
4	100	400	100	400	r,d,dd,na,g
5	120	1700	101	1700	r,d,dd,na,rns

[r: RNAi abstracts, d: drug only abstract, dd: drug interaction abstracts, na: not applicable, rns: random negative set]

### Selection of algorithm

Evaluation is key to identifying the best classifier that can perform the given task with the highest accuracy. With the limited amount of data for training and testing, the 10 fold stratified cross validation was chosen as the most appropriate method for evaluating the various classifiers. The dataset was evaluated using the following 7 classifiers, namely, ZeroR, NaiveBayes, K-nearest neighbor, J48, Random Forest, Support Vector Machine and OneR. These are some of the most commonly used algorithms for text classification, except for ZeroR which was used here to get a baseline. The filtered classifier belonging to the WEKA [32] meta classifier was used, since it has the advantage of simultaneous selection of a classifier and filter to evaluate the model. The various classifiers mentioned above were tested along with the string to word vector filter. The string to word filter converts string attributes into a set of attributes that represent the word occurrences from the text contained within the strings. The set of attributes is determined from the training data set. The 10 fold stratified cross validation option was selected and the data from the training set (Table 1) was evaluated to identify the best classifier.

### Training and testing the model

Based on the classification accuracy of the above 5 models, the top three were selected for training and testing These models were trained and then tested on the dataset shown in Table 1. The highest performing model namely SMO trained on Set 4 (SMO-4) was chosen as the model to be used on the unknown dataset. The model was further improved by adding a randomly generated set, to improve the classification of abstracts. A random number generating script was used to randomly select 10,000 numbers between 10,000,000 and 25,000,000. The numbers thus obtained were used as PMIDs to download the respective abstracts. These abstracts were processed and converted to the attribute relationship file format. The 10,000 abstracts were tested using the SMO-4 model. The abstracts that were classified as RNAi by SMO-4 were eliminated. The remaining abstracts formed the random negative dataset. This step ensures that the random negative set is free of positive RNAi instances. The randomly generated dataset was included in the dataset 5. The dataset shown in Table 1 was used to evaluate a new model using the filtered classifier (SMO/StringToWordVector) and named as SMO-5. The performance of SMO seemed to be better and consistent and was chosen as the model of choice for further analysis.

### Generation of the screening dataset

The abstracts for the years 1975 – 2015 was downloaded from the MEDLINE database. The abstracts were downloaded and converted to individual text files retaining just the PMID, title and abstract text. The text files were grouped by year and then converted to the attribute relationship file format using the WEKA TextDirectoryLoader class. The individual .arff weka input files were updated to reflect the classes that were used to generate the classification model (SMO-5), namely RNAi and Non_RNAi.

### Extraction of RNAi relevant abstracts

The weka arff files containing the abstracts for each year from 2001 to 2015 was classified using the SMO-5 classification model on the Bigred2, a Cray XE6/XK7 supercomputer with a hybrid architecture comprising of 1020 computing nodes. A total of 10.5 million abstracts were processed to be classified as RNAi or Non_RNAi. The resultant file containing the PMID’s along with the classification as RNAi or Non_RNAi was further processed to extract the PMIDs of abstracts classified as RNAi. The abstracts for these PMID’s were retrieved and converted to XML format retaining the PMID, article title and abstract text.

### Creation of dictionary for entity recognition

A perl module was created to house the dictionaries for gene names and cell line names. The list of gene names along with their aliases was downloaded from HGNC (HUGO Gene Nomenclature Committee) [33] and the list of cell lines names along with their aliases was downloaded from cellosaurus [34]. These list were further processed to form the final dictionary with cell line names and gene names normalized to their official names/symbols. These dictionaries are very comprehensive with the Gene dictionary containing 161,863 entries and the cell line dictionary containing 73,370 entries.

### Entity tagging and cell-gene information extraction

The abstracts that were classified as RNAi were further processed and the gene and cell line mentions were tagged with the normalized name of the cell line or gene name using the dictionary that was created as mentioned above. Once tagged the abstracts were further processed to extract the cell line name and gene names. These were stored in a table format to preserve the genes studied in a given cell line within a given abstract.

### Validation of the essential genes

The extracted genes were ranked in descending order of number of studies associated. The genes that were studied on an average of 100 or more times were extracted and the cell lines in which these genes were studied on average of 20 or more times were extracted as well. In addition the top 20 most studied genes, the median 20 genes and the bottom 20 genes were extracted. The correctness of the extracted cell gene associations was verified by selecting the relevant PMIDs and manually scanning for the presence of the cell and gene information that was extracted. The top genes predicted to be essential for cell survival was queried against the network of cancer genes [35] to identify their relevance to cancer and were also queried against the Therapeutics Target Database [36] to identify if they were drug targets. The genes were also queried against the DPSC database [37] at a threshold *p*-value of <0.05 to check for them being reported as essential genes.

## Results

### Identification of siRNA relevant abstracts and corpus creation

From the approximately 2000 abstracts that were manually screened 221 belonged to the RNAi class and 1644 belonged to the Non_RNAi class. The Non_RNAi class included abstracts from drug, drug-drug or the not applicable class as described in the methods section. The average inter classification agreement among individuals who manually read the abstracts was 0.75.

Since these abstracts were initially downloaded based on the specific cell lines prior to the manual scan, there were duplicate abstracts among the cell lines. Following the manual classification task, the entire dataset was scanned for duplicate PMIDs and they were removed. In order to get a better representative negative set, the randomly generated dataset as mentioned in the methods section which consisted of 10,000 abstracts was created. Thus creating a dataset that had a wider coverage than just the ones that were picked during the initial screening. The above mentioned datasets formed the text corpus to be used for RNAi text classification. This dataset was further divided into training and testing data for evaluating and training the models for RNAi text classification.

### Evaluation of the classifiers

In order to get an estimate of the generalization error each of the classifiers chosen was evaluated using the 10 fold stratified cross validation. The classifiers were evaluated and the results as percent correctly classified are as shown in Table 2. The zeroR classifier is used here to determine the baseline performance and as a benchmark for the other classification methods used. The zeroR classifier is the simplest classification method and does not have any predictability power. It simply builds a frequency table of the given data and selects the most frequent value as its prediction. It can be noted from the Table 2 for zeroR that the percentage accurately predicted is the same as the percentage of the class that is most abundantly present. From Table 2, it can be observed that the composition and balance between the positive and negative set does affect the accuracy results of some of the classifiers. Overall the J48, NaiveBayes and SMO seemed to be consistent across the various datasets and more immune to the varying changes between the dataset size and composition.

**Table 2:** The % accuracy of classification after evaluating each classifier on a given dataset using 10 fold stratified cross validation

Classifiers	Set 1	Set 2	Set 3	Set 4	Set 5
ZeroR	75.00	50.00	75.00	80.00	93.41
NaiveBayes	93.00	89.00	93.25	92.40	95.00
KNN	77.00	74.00	81.00	83.20	94.23
J48	95.00	95.00	94.50	96.60	98.46
RandomForest	91.00	95.00	84.75	82.80	93.41
SMO	94.25	94.50	94.50	96.00	98.35
OneR	88.75	78.00	88.75	91.00	96.09

### Evaluating the performance of the top 3 models

The top 3 classifier models with the highest accuracy of prediction for a given dataset was chosen for further analysis to determine the final model to be selected for RNAi text classification. Each of the top 3 performing models evaluated on a given dataset was further trained on the respective datasets that were used for their evaluation in the 10 fold stratified cross validation, following which they were tested on a previously unseen dataset, namely the test dataset. The performance results from training and testing are as shown in Table 3.

**Table 3:** The % accurately classified by the top three models after training and testing

Set 1	Train	Test	Set 2	Train	Test	Set 3	Train	Test
J48	99.50	94.50	J48	99.00	93.00	J48	99.50	94.50
SMO	100.00	96.25	RandomForest	100.00	97.50	SMO	100.00	94.50
NaiveBayes	98.00	86.50	SMO	100.00	93.00	NaiveBayes	98.50	84.25
Set 4	Train	Test	Set 5	Train	Test			
J48	99.00	92.40	J48	99.50	99.20			
SMO	100.00	93.00	SMO	100.00	98.50			
NaiveBayes	94.20	89.00	oneR	96.60	97.10			

These models were previously evaluated using the 10 fold cross validation

In addition to the performance measures such as percent correctly classified, precision and recall, using the error rate is a good way of measuring the classifiers performance. It can been seen from Table 4 that J48 and SMO have the best performance according to the five error metrics. They have the lowest values for the mean absolute error, root mean squared error, relative absolute error and root relative squared error and the highest value for the kappa statistic making them the models of choice.

**Table 4:** Classifier errors for the classifier’s tested on dataset 5

Classifier Error	ZeroR	NaiveBayes	KNN	J48	RandomForest	SMO	OneR
Kappa statistic	0.00	0.66	0.23	0.87	0.00	0.86	0.57
Mean absolute error	0.12	0.05	0.06	0.02	0.09	0.02	0.04
Root mean squared error	0.25	0.22	0.24	0.12	0.20	0.13	0.20
Relative absolute error	100%	40.55%	47.10%	15.63%	73.11%	13.33%	31.55%
Root relative squared error	100%	90.13%	96.73%	48.74%	79.35%	51.73%	79.59%

**Fig. 1:**
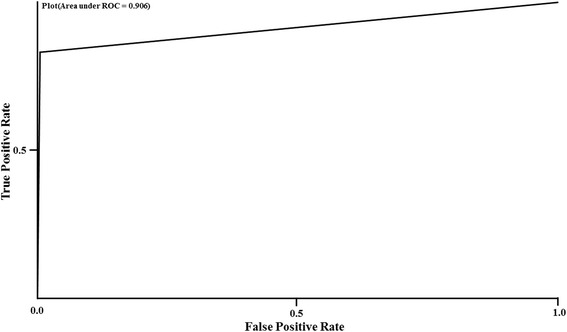
AUC Receiver Operator Characteristics for the SMO-5 model

It can be noted that J48 and SMO performed the best. Since SMO was consistently better across the various datasets and SVM being a preferred, faster performing and reliable classifier for text classification, it was chosen for further analysis. The various performance metrics for abstracts classified as RNAi are shown in detail for the classifiers tested on dataset 5 in Table 5 and the classifier errors are shown in Table 4. The J48 and SMO models performed the best with the SMO model being faster in time taken to build the model. In addition the ROC curve (Fig. 1) for the SMO-5 proves its efficiency as a very good classification model.

**Table 5:** Performance metrics across the various classifiers tested on dataset 5 for abstracts classified as RNAi

Classifiers	Time (sec)	TPR	FPR	Precision	Recall	F-Measure
ZeroR	2.45	0.00	0.00	0.00	0.00	0.00
NaiveBayes	28.82	0.83	0.04	0.59	0.83	0.69
KNN	3.22	0.14	0.00	0.90	0.14	0.25
J48	116.14	0.83	0.00	0.93	0.83	0.88
RandomForest	70.66	0.00	0.00	0.00	0.00	0.00
SMO	6.46	0.82	0.01	0.93	0.82	0.87
OneR	12.94	0.42	0.00	0.98	0.42	0.59

[Time in seconds to build the model, True Positive Rate (TPR), False Positive Rate (FPR)]

### Genes essential for cancer cell survival

A total of 10.5 million abstracts from the years 2001 to 2015 were tested using the SMO_5 model which resulted in 32,164 abstracts being classified as RNAi (Table 6). These abstracts spanned over 1467 cancer cell lines and 4373 genes. There was a total of 25,891 cell gene associations identified (Table 7), out of which 97% of the associations between a cell line and a gene occurred 5 or less times. Only 2 gene-cell line pairs were studied more than 90 times. Among the 1467 cell lines 88% of them had at least 1 or up to 25 genes studied in a given cell line (Table 8). Among the 4373 genes 96% of them were studied in at least 1 or up to 25 different cell lines (Table 9).

**Table 6:** The number of abstracts that were processed per year and the number of abstracts that were identified as relevant to RNA interference studies

Year	Medline	RNAi
2001	424,042	101
2002	435,427	180
2003	472,745	425
2004	514,910	745
2005	575,403	1101
2006	620,688	1503
2007	652,232	1724
2008	701,623	1996
2009	742,510	2308
2010	801,061	2707
2011	862,838	3070
2012	931,619	3923
2013	978,796	4048
2014	1,018,012	4498
2015	796,876	3835
Total	10,528,782	32,164

**Table 7:** The number of times a given gene and cell line were studied together

No. of Cell Gene Associations	Frequency
5	25,198
10	461
15	99
20	52
25	25
30	15
35	8
40	4
45	10
50	1
55	1
60	6
65	5
70	1
75	2
80	0
85	1
90	2

**Table 8:** Frequency of the number of genes studied in a given cell line

Genes	Frequency
25	1291
50	73
100	54
200	30
300	10
400	3
500	1
600	2
700	0
800	1
900	1
1000	0
1100	1

**Table 9:** Frequency of the number of cell lines used to study a given gene

Cell Lines	Frequency
25	4209
50	96
100	46
150	10
200	5
250	3
300	1

**Fig. 2:**
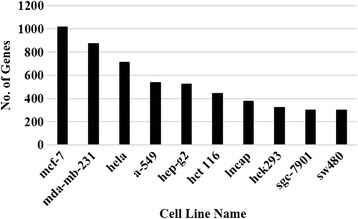
Top 10 most studied cell lines

**Fig. 3:**
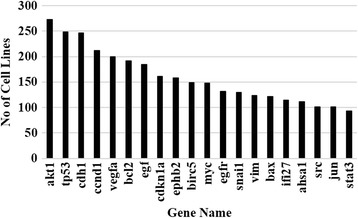
The top 20 genes predicted to be essential for cell survival

The top 10 cell lines extracted namely, MCF7, MDA-MB-231, HELA, A549, HEPG2, HCT116, LNCAP, HEK293, SGC7901 and SW480 (Fig. 2) had on an average 300 or more associated gene studies and represented Breast, Lung, Colon, Gastric, Liver, Cervical, Prostate and Kidney cancers, which are some of the most common cancers that affect men and women. On analyzing the cell lines and genes extracted from these abstracts, the top 20 genes, namely AKT1, TP53, CDH1, CCND1, VEGFA, BCL2, EGF, CDKN1A, EPHB2, BIRC5, MYC, EGFR, SNAI1, VIM, BAX, IFI27, AHSA1, SRC, JUN and STAT3 had on an average 100 studies or more associated across different cell lines as shown in Fig. 3. Among the top 20 genes, 9 of them are known cancer genes that have a role in cellular function as shown in Table 10 [35]. These functions are defined in the biological process branch of the Gene Ontology (GO) levels 5 and 6. Out of the top 20 genes queried against the DPSC database, 15 of the genes were found to be essential among the four cancer types, namely breast, colon, ovarian and pancreas. In addition 11 out of the 20 genes have active drugs that are being studied in clinical trials or being researched as a potential therapeutic target, some of which have been approved. (Additional file 1: Table S1) [36].

**Table 10:** The genes amongst the top 20 that are known to be cancer genes and their roles in the various processes required for cellular function

Functional Class	AKT1	TP53	CDH1	CCND1	BCL2	CDKN1A	MYC	EGFR	JUN
Cell cycle	X	X		X	X	X	X	X	
Cell motility and interactions			X					X	
Cell response to stimuli	X	X		X	X	X			
Cellular metabolism	X	X		X		X	X	X	X
Cellular processes	X	X	X	X	X			X	X
Development	X	X		X	X	X		X	X
DNA/RNA metabolism and transcription		X					X		X
Immune system response	X				X	X			
Multicellular activities	X				X				
Regulation of intracellular processes and metabolism	X	X	X	X	X	X	X	X	X
Regulation of transcription	X	X					X		X
Signal transduction	X	X		X	X	X		X	

X: genes involved in that particular functional process of the cell

The top 20 genes studied on an average 20 or more times in a given cell line was extracted and the cell lines were associated to their respective cancer types. The number of genes among the top 20 genes that are associated with a given cancer type is shown in the Additional file 1: Table S2. All of the top 20 genes were studied in breast cancer, indicating the complexity of this disease and the network of genes that may play a role in the progression of this cancer.

### Validation of genes predicted to be essential

The top 20 genes, the median 20 genes and bottom 20 genes were extracted and were manually verified from the respective abstracts for their essentiality in cell survival. The top 20 genes were all found to be essential towards cell survival. Among the median 20 there were around four that were false positives and among the bottom 20 there were two that were false positives and four that were genes found to be essential in a non-human species (Additional file 1: Table S3).

## Discussion

In multicellular organisms, cell death is a critical process by which the damaged cells or those that pose a threat to the organism are destroyed through a tightly regulated process of cell destruction [38–40]. This process is very essential for the overall health and survival of the organism as it gets rid of the cells that may interfere with its normal function [41]. It is clear that a crucial balance between cell proliferation and cell death should be maintained and tipping to one side could lead to a diseased state. Cancer, the uncontrolled proliferation of cells is one of the most complex and challenging disease to treat as it involves many underlying molecular mechanisms and moreover these mechanisms are shared alike by cancerous as well as normal cells. This sharing makes it difficult to therapeutically target cancerous cells without damaging the normal cells. Most of the chemotherapeutic agents available today are relatively nonspecific and cause considerable damage to the surrounding normal cells, leading to severe adverse events. Thus identifying those molecular mechanisms that are essential only to the survival of cancerous cells but not normal cells holds the key to effective cancer treatments. In addition the heterogeneity of cancer calls for a systematic identification of genes that are essential for the growth of these diverse set of cells and the resultant cancer phenotype which can aid in the identification of potential drug targets.

Our top hit, AKT is a major signaling hub for various downstream substrates and is known to be critical for cell growth and survival [42–44]. It is involved in the progression of many human cancers [45–47]. There are various therapeutic interventions that are currently being targeted towards the inhibition of AKT [48–50]. Perifosine, MK-2206, RX-0201, PBI-05204 and GSK2141795 are some of the potential AKT inhibitors being investigated in several cancers [50].The role of AKT in promoting cell proliferation and survival in hormone responsive MCF-7 breast cancer cells has been previously studied [51]. The investigational drug, MK-2206 has been found to be effective in treating breast cancer [52]. It has been shown that increased levels of AKT in certain cell lines is associated with acquired resistance to antiestrogenic therapy and an inhibition of AKT led to a pronounced growth inhibition of the cell lines [53]. With a wide array of involvement in cell survival and cancer progression, AKT is a potential drug target in cancer therapy, yet finding an optimal way to inhibit AKT has been elusive. Identifying the genes that are essential for cell survival and those that drive tumor resistance are critical pieces of information for developing targeted therapies to prevent the progression of cancer.

p53 has been widely studied and is best known for its tumor suppressing ability through the initiation of apoptosis. The p53 gene once hailed as a potential therapeutic target to halt cancer is met with complexity as many of its functions remain unclear. It’s ability to regulate the same cellular processes both positively and negatively makes it hard to predict the outcomes of its activation [54].

Moreover, the median 20 and bottom 20 genes, though not frequently studied may hold the answers to treating cancers that respond poorly to therapy. For example the NFAT gene from our bottom 20 gene list has been found to be involved in many solid tumors and malignancies [55–57]. This and many other genes extracted during this process can be exploited for their role in cancer.

Most of the top essential genes identified and extracted through the large scale scanning of PubMed abstracts are involved in the survival pathways and in various malignancies – AKT1 [48–50, 53, 58–62], TP53 [54, 63, 64], CDH1 [65, 66], CCND1 [67], VEGFA [68, 69], BCL2 [70, 71], ITK [72], CDKN1A [73], EPHB2 [74, 75], BIRC5 [76], MYC [77], EGFR [78, 79], VIM [80, 81], BAX [82], AHSA1 [83], and SRC [84]. This suggests that the growth and survival of cancer cells is sustained by a network of genes that come into harmony to fuel the cancer progression. This clearly brings out the importance in not only targeting essential genes, but also those that may be closely involved but not very evident as to their role in fueling cancer. This calls for an extensive mining of data and literature in search of genes that are less known but critical in cellular processes, as these could play a crucial role in the progression of complex disease just as rare SNPs do. The co expression of a gene may not mean that it is or has an influence on the essential gene identified here. But it could mean that in the absence of the targeted essential gene, the co-expressed gene could possible play a role in promoting cell survival, a fact that cannot be ruled out. The complexity of effectively treating cancers unfolds as the network of genes linked to essential genes grow. Identifying the potential interaction that exists between these genes and their individual roles in cell survival or the extent of their influence within a pathway can shed light into developing targeted therapies that destroy cancerous cells but leave the normal cells intact.

There are a few limitations to the method used here. Even though majority of the genes found to be essential are identified and associated with their respective cancer cell lines, there have been instances where a gene or gene alias was the same as that of a commonly used word in English and got tagged incorrectly leading to a false positive. Another limitation of this process is that it cannot identify instances where a gene was specifically found to be not essential for a given cell line.

## Conclusion

It is very evident thus far that the efficacy of a therapeutic intervention is multifactorial in nature and in many cases the source of therapeutic disruption could be from an unsuspected source. This approach in scanning millions of abstracts to identify top genes that are essential for survival is a feat that is not possible by an individual researcher or a group, just because of the sheer volume of literature that needs to be processed and the connections between entities to be made. Using machine learning algorithms, has not only helped narrow down the search and provided information about essential genes in different cancer types but also provided the building blocks to generate a network of interconnected genes and processes, which can be used to generate hypothesis that can be experimentally validated to improve our understanding of what triggers and maintains the growth of cancerous cells. This comprehensive list of genes that are predicted to be essential in various cancer types can be used as an informational tool by researchers who wish to identify more genes that may be crucial to answer the questions they may have in treating a specific type of cancer. Moreover when the top essential genes do not provide all the answers that a research is seeking, they can expand their targeted gene list by utilize this resource to look up the less frequently studied genes which might prove to be more critical just as rare variants are in finding answers to treating complex diseases. Since genes that are essential are typically involved in biological processes that are critical to a cell, the identification of essential genes in other species through this process can be used as a method of identifying novel targets that would have otherwise gone unnoticed.

## Additional file

Additional file 1: The file contains three sheets labelled Table S1 - S3. The tables list the genes that are currently targeted for treating various cancers, the number of top 20 genes that were studied in a given cancer type and the gene- cell associations that were manually verified. (XLSX 16 kb)

## Abbreviations

ARFF: Attribute Relation File Format
DPSC: Donnelly - Princess Margaret Screening Centre
HGNC: HUGO Gene Nomenclature Committee
RNAi: RNA interference
RNS: Random Negative Set
ROC: Receiver Operating Characteristic
shRNA: Small(or short) hairpin RNA
siRNA: Small(or short) interfering RNA
SMO: Sequential Minimal Optimization
SVM: Support Vector Machine
WEKA: Waikato Environment for Knowledge Analysis
